# Discovery and characterization of complete genomes of 38 head-tailed proviruses in four predominant phyla of archaea

**DOI:** 10.1128/spectrum.00492-24

**Published:** 2024-11-15

**Authors:** Tianqi Xu, Yimin Ni, Hailing Li, Shuang Wu, Shuling Yan, Lanming Chen, Yongxin Yu, Yongjie Wang

**Affiliations:** 1 College of Food Science and Technology, Shanghai Ocean University, Shanghai, China; 2 Entwicklungsgenetik und Zellbiologie der Tiere, Philipps-Universität Marburg, Marburg, Germany; 3 Laboratory for Marine Biology and Biotechnology, Qingdao Marine Science and Technology Center, Qingdao, China; NC State University, Raleigh, North Carolina, USA

**Keywords:** archaeal provirus, virus diversity, virus–host interaction

## Abstract

Archaea play a significant role in natural ecosystems and the human body. Archaeal viruses exert a considerable influence on the structure and composition of archaeal communities and their associated ecological environments. The present study revealed the complete genomes of 38 archaeal head-tailed proviruses through comprehensive data mining. The hosts of these proviruses were identified as belonging to the following four dominant phyla: *Halobacteriota*, *Thermoplasmatota*, *Thermoproteota*, and *Nanoarchaeota*. In addition to the 14 proviruses of halophilic archaea related to the *Graaviviridae* family, the remaining proviruses exhibited limited genetic similarities to known (pro)viruses, suggesting the existence of 14 potential novel families. Of the 38 archaeal proviruses, 30 have the potential to lyse host cells. Eleven proviruses contain genes linked to antiviral defense mechanisms, including those involved in restriction modification (RM), clustered regularly interspaced short palindromic repeat (CRISPR)-associated (CRISPR–Cas) nucleases, defense island system associated with restriction-modification (DISARM), and DNA degradation (Dnd). Moreover, auxiliary metabolic genes were identified in the proviruses of *Bathyarchaeia* and *Halobacteriota* archaea, including those involved in carbohydrate and amino acid metabolism. Our findings indicate the diversity of archaeal viruses, their interactions with archaeal hosts, and their roles in the adaptation of the host.

**IMPORTANCE:**

The field of archaeal virology has seen a rapid expansion through the use of metagenomics, yet the diversity of these viruses remains largely uncharted. In this study, the complete genomes of 38 novel archaeal proviruses were identified for the following four dominant phyla: *Halobacteriota*, *Thermoplasmatota*, *Thermoproteota*, and *Nanoarchaeota*. Two families and six genera of Archaea were the first to be identified as hosts for viruses. The proviruses were found to contain diverse genes that were involved in distinct adaptation strategies of viruses to hosts. Our findings contribute to the expansion of the lineages of archaeal viruses and highlight their intricate interactions and essential roles in enabling host survival and adaptation to diverse environmental conditions.

## INTRODUCTION

Archaea are a widely distributed group of prokaryotes. Initially, archaea were believed to inhabit only extreme environments, such as hot springs and salt lakes. However, by the late 20th century, archaeal communities had been identified in a range of diverse ecological environments (1). Archaea play a significant role in a multitude of ecosystems and have demonstrated considerable potential in the field of biotechnology. The discovery of novel metabolic pathways, particularly in methanogenic archaea, has facilitated the advancement of archaeal applications (2, 3). Additionally, archaea function as symbiotic microorganisms that colonize human organs, with a prevalence in the respiratory, oral, and gastrointestinal tracts (4–7).

The advancement of high-throughput sequencing and automated analysis processes is facilitating the accelerated discovery and exploration of viruses. The majority of known viruses have been isolated and identified in halophilic and thermophilic archaea, primarily in the *Euryarchaeota* and *Crenarchaeota* phyla (8). Recent metagenomic studies have revealed the diversity of archaeal (pro)viruses within *Thaumarchaeota* (9–13), methanogenic archaea (6), and Asgard archaea (14–16). Furthermore, multiple *Methanobrevibacter_A* archaeal proviruses have been identified within the human gut microbiota (6, 7). Archaeal proviruses are defined as viral genomes that have integrated into the genomic DNA of the host cell (17). The majority of known proviruses are head-tailed viruses and pleolipoviruses, which are a group of pseudo-spherical and pleomorphic archaeal viruses composed of a membrane vesicle enclosing a DNA genome (ICTV, https://ictv.global/report/chapter/pleolipoviridae/pleolipoviridae). These proviruses are mainly associated with hosts from the *Halobacteriota*, *Methanobacteriota*, and *Thermoproteota* phyla (18, 19). At present, several archaeal proviruses are being induced, including the *Aeropyrum pernix* spindle-shaped virus 1 (APSV1) and the *Aeropyrum pernix* ovoid virus 1 (APOV1) (18). Two temperate viruses were isolated from halophilic archaea: the icosahedral SNJ1 and the polymorphic virus SNJ2 (20). Sequence analysis revealed the presence of diverse archaeal proviruses, indicating their potential significance in virus–host interactions (20).

The discovery of archaeal viruses continues to present significant challenges for researchers in the field. At present, the International Committee on Taxonomy of Viruses (ICTV) has catalogued 135 complete genomes of archaeal viruses, which have been divided into 15 orders and 46 families. These figures are considerably lower than those observed for bacteriophages. A further significant challenge is the identification of viral hosts. Despite the use of non-culture-dependent methods, such as CRISPR spacer–protospacer targeting, tRNA matching, and deep learning techniques (21, 22), host identification in metaviromics remains a highly challenging issue.

Previous investigations into archaeal viruses have contributed to a deeper understanding of their diversity. For example, a metavirome of the East China Sea (ECS) surface waters identified sequences affiliated with uncultivated marine *Thaumarchaeota* dsDNA viruses and Magroviruses (23, 24), and a set of 36 novel archaeal viruses (25, 26).

This study reports the discovery of 38 novel archaeal proviruses in publicly available archaeal genomic databases. The discovery was made using the major capsid proteins (MCPs) of archaeal viruses found in our recent study (25), along with two typical genes of integrase and tRNA that are involved in integration sites (27–30). The results contribute to our knowledge of the diversity of archaeal viruses and their interactions with hosts.

## MATERIALS AND METHODS

### Archaea genome database and classification

The archaeal databases used in this study were sourced from the National Center for Biotechnology Information (NCBI) Archaea metagenomic assembly genomes as of 13 May 2022. Sequences longer than 10 kbp were filtered using Seqkit v2.3.0 (parameters: seq -m 10000) (31), yielding a data set comprising 303,395 contigs, including 1,968 complete archaeal genomes. The Archaea were classified using the default settings of the GTDB taxonomy identification software (GTDB-TK v2.1.1) (32, 33).

### Identification of archaeal proviruses

**Fig 1:**
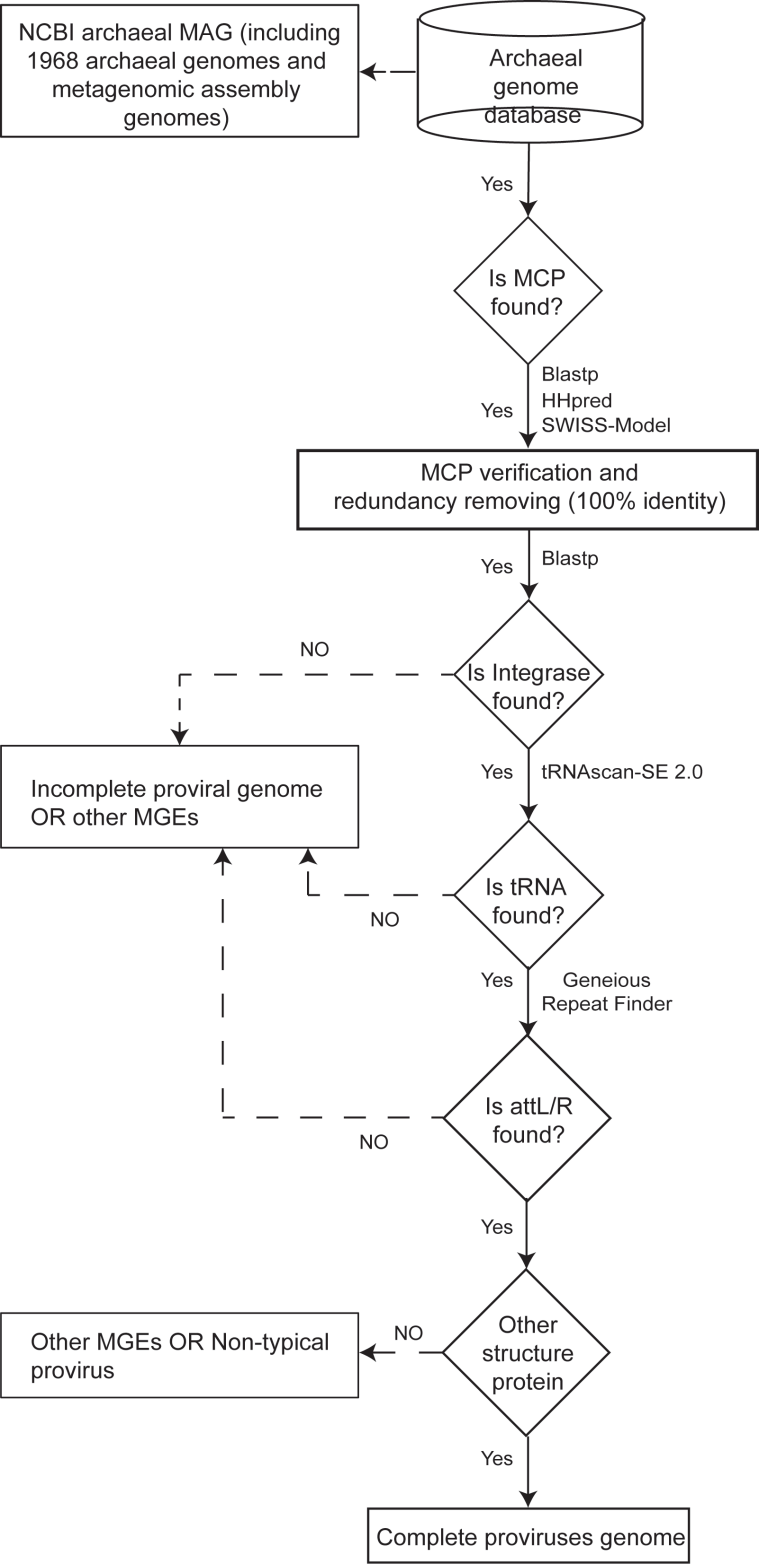
Pipeline for identifying archaeal proviruses.

The pipeline for the identification of archaeal proviruses is shown in Fig. 1. The iterative PSI-BLAST search against the archaeal genomic sequence database used 30 major capsid protein (MCP) sequences (Table S1) of Yangshan Harbor (YSH) new archaeal viruses from our previous study as seeds (E-value <1e−5, identity >30%, and coverage >50%) (25). In each iteration, newly identified MCP hits were validated using BLASTp (with an E-value <1e−5) against the non-redundant (nr) protein database (Release 255, 15 April 2023) and HHpred with default parameters against the Pfam-A_v35, PDB_mmCIF70_18_June, COG_KOG_v1.0, PGROGs_v4, and SWISS-MODEL (34–36).

The integrase gene is typically found in close proximity to the integration site, marking one end of the integrated proviruses (28), and viral integrase-catalyzed insertion frequently results in the duplication of the integration site, which then flanks the provirus as a direct repeat (29). Moreover, bacterial and archaeal viruses have been observed to target the 5′- or 3′-distal regions of tRNA genes for integration (27, 30). Consequently, to identify complete provirus sequences, the upstream and downstream regions of the MCP genes identified in the archaeal genomic sequences were analyzed for these two genes and potential attachment sites (attL/R) (30) . The archaeal sequences containing the MCP were analyzed using the software tool Prodigal v2.6.3 (parameter: -p meta) (37) to predict encoded proteins. The predicted proteins were then annotated using BLASTp against the non-redundant protein database with an E-value threshold of less than 1e−5. The Geneious Prime’s Repeat Finder plugin was used to identify direct repeats (DRs), while tRNA genes were predicted using tRNAscan-SE 2.0 with the parameters set to −A (38, 39).

### Protein-based gene-sharing network of archaeal proviruses

The gene-sharing network analysis was conducted using vConTACT v2.0 (40). The input sequences included 38 archaeal provirus sequences discovered in this study, archaeal virus sequences from IMG/VR4 (41), and recently published archaeal (pro)viruses from the literatures (12, 42–46). Viral protein sequences were predicted using Prodigal v2.6.3 (parameter: -p meta) (37). The sequences were then mapped to a CSV file using vConTACT2’s gene_to_genome program. The nucleotide, protein, and CSV files were input into vConTACT2 using the following parameters: -r --rel-mode Diamond -p --db None --pcs-mode MCL --vcs-mode ClusterONE. The gene-sharing analysis results were imported into Cytoscape v3.10.0 to generate the viral gene-sharing network diagram (47).

### Protein function annotation of archaeal proviruses

Protein sequences of proviruses were predicted using Prodigal v2.6.3 (parameter: -p meta) (37). Protein functions were annotated by performing BLASTp (E-value <1e−5) against the nr protein database (35). Functional prediction of conserved domains was performed using batch CD-Search with default parameters against the conserved domain database (CDD) (48). COG functional category of ORFs was analyzed using COGclassifier with default parameters (49). For proteins with unknown functions, a more sensitive Markov chain model was employed for functional annotation. HHpred was used for profile–profile comparisons against the databases of Pfam-A_v35, PDB_mmCIF70_18_June, COG_KOG_v1.0, and PGROGs_v4 (36, 50).

### Prediction of antiviral defense system in proviral genomes

The Prokaryotic Antiviral Defense Locator v2.0.0 tool (PADLOC v2.0.0) (51, 52), accessible via https://padloc.otago.ac.nz/padloc, was employed to predict the antiviral defense systems within proviral genomes. The CRISPR/Cas defense system was predicted by utilizing the online version of CRISPRCasFinder (53) with the subsequent parameter settings of Allow Repeat Mismatch, Perform CAS gene detection, and selecting the SubTyping clustering model. The other parameters were set to its default values.

### Prediction of lytic potential of archaeal proviruses

The lytic potential of archaeal proviruses was predicted using the PhaTYP server with default parameters (54). If the genomes contained genes associated with lysis, such as holin and endolysin genes, they were considered to possess lytic potential (55, 56).

### Prediction of auxiliary metabolic genes of archaeal proviruses

Auxiliary metabolic genes (AMGs) of archaeal proviruses were predicted based on the VIBRANT-1.2.0 tool on the CyVerse platform, with parameters set as -Length in basepairs 1000, -Number of ORFs 4, and -virome True (57). Subsequently, the predicted AMGs were further annotated and confirmed through HHpred against the Pfam-A_v35, PDB_mmCIF70_18_June, COG_KOG_v1.0, and PGROGs_v4 databases (36, 50).

### Phylogenetic analysis

The viral proteomic tree was generated using the ViPTree web server v3.0 with default parameters (58). The input sequences consisted of 37 complete genomes of proviruses identified in this study and six related head-tailed archaeal proviruses. The database was the archaeal virus reference database.

The phycocyanobilin lyase subunit alpha (CpcE) identified in an archaeal provirus was searched for homologous proteins in the NCBI nr database using BLASTp with default parameters (35). The top hits were selected and subjected to multiple sequence alignment using MAFFT (parameter: --maxiterate 1000 --globalpair) (59). Columns in the alignments with more than one-third of gaps were removed using trimal v1.4 (parameter: -gt 0.3), and single-gene phylogenetic tree was constructed using FastTree v2.1.12 (parameter: -wag -gamma) (60, 61).

### Calculation and visualization of orthologous fraction (OF) of archaeal proviruses

The average amino acid identity (AAI) of orthologous fraction (OF) between virus genomes was calculated using the compareM tool (https://github.com/dparks1134/CompareM). The input sequences were the same as those used in the above ViPTree analysis. The parameters used were -evalue 1e-5 and -identity 30%. The results were visualized with heatmap using tidyr v1.3.0 and pheatmap v1.0.12 (R version 4.2.1).

## RESULTS AND DISCUSSION

### Identification of archaeal proviruses

**Fig 2:**
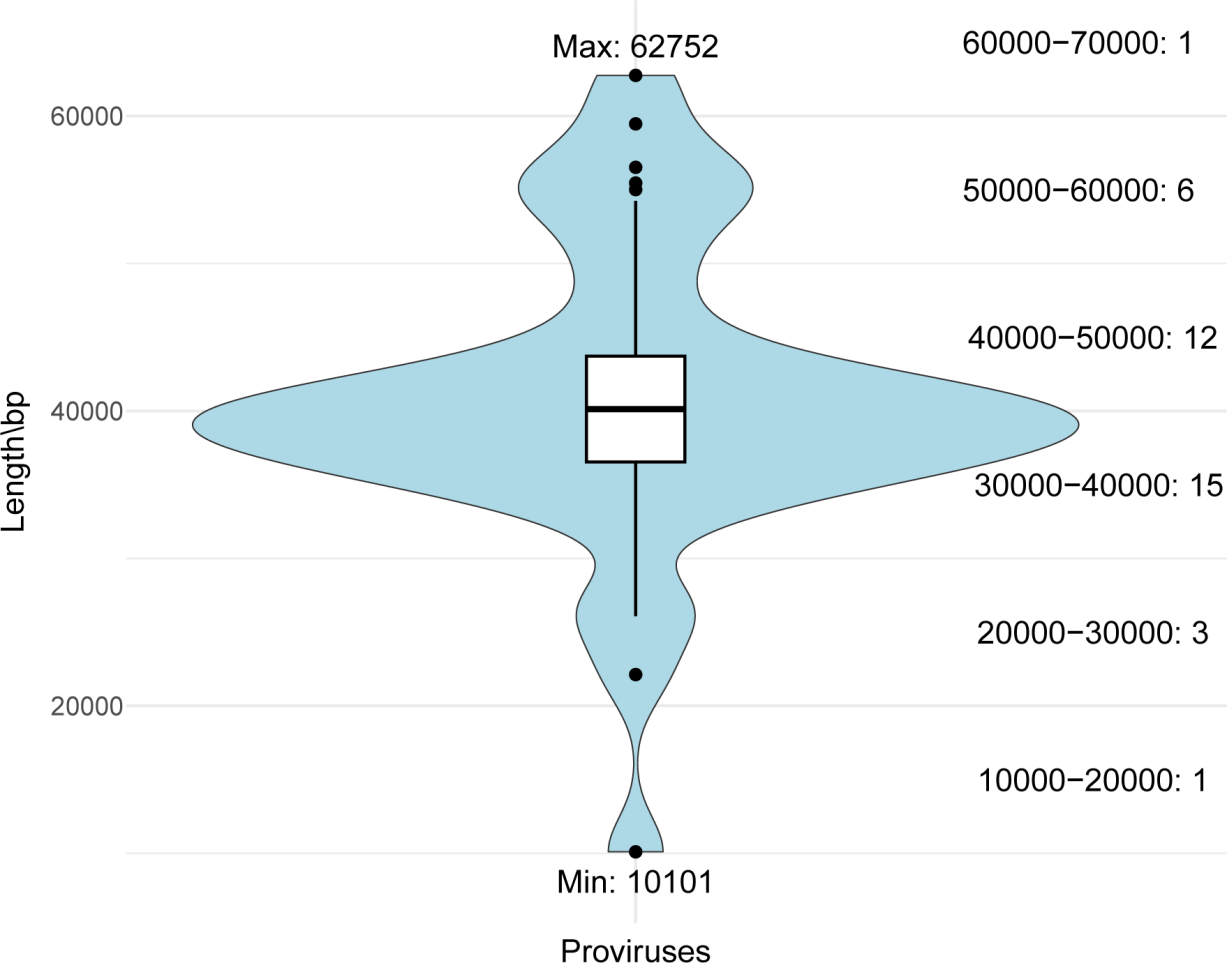
The genome length range of 38 proviruses. The numbers on the right denote both the range of genome lengths and the count of proviruses falling within each range.

A total of 710 major capsid proteins (MCPs) were identified in the archaeal genomic database, and the corresponding sequences were extracted. Following the removal of redundant sequences (100% identity), 629 archaeal sequences were retained (Table S2). To identify complete provirus genomes, it was necessary to consider a number of factors, including the MCPs and other genome boundary markers, such as the integrase gene, DR, and tRNA gene (Fig. 1). Ultimately, a final set of 58 complete provirus genomes was identified. Of these, 38 sequences were identified as novel and underwent further analysis (Table S3), exhibiting a size ranging from 10.0 to 62.7 kbp (Fig. 2). The remaining 20 were identical to those previously reported in references (7, 42, 44, 62), thereby providing further evidence to support the reliability of the methods employed for the identification of archaeal proviruses.

To determine the hosts of the 38 proviruses, a taxonomic classification was carried out utilizing GTDB-TK v2.1.1 (32). The majority of the 38 proviruses were identified in organisms belonging to the phylum *Halobacteriota* (Table S3). Eight proviruses were hosted by organisms belonging to the class *Bathyarchaeia* of the phylum *Thermoproteota*, two proviruses were classified as belonging to the order *Woesearchaeales* of the phylum *Nanoarchaeota*, and one provirus had a host in the phylum *Thermoplasmatota* (Table S3). Proviruses have been identified in several archaeal phyla, including *Halobacteriota*, *Thermoproteota*, *Methanobacteriota*, and *Thermoplasmatota* (7, 12, 18–20, 29, 42–46). However, these proviruses exhibit a distinct phylogenetic divergence from the 38 proviruses under discussion in this study, indicating that a substantial reservoir of archaeal proviruses remains to be explored within the confines of the currently available archaeal sequences. Importantly, no virus was identified in the two families of proviral hosts, specifically the DTDX01 family in *Thermoproteota* and the B72-G16 family in *Nanoarchaeota*, as well as in the six genera of proviral hosts, comprising four *Bathyarchaeia* archaea and two *Halobacteriota* archaea (Table S3). The absence of evidence for viral infection in archaea may be attributed to the presence of novel antiviral defense systems within these archaea or the emergence of novel viruses that are challenging to detect through homology searches or chronic infections without cell lysis (63).

It should be noted that the exclusion of the remaining 571 archaeal sequences from further analysis does not imply that they are devoid of value; rather, it is a decision based on considerations of data reliability and novelty, as well as the need to facilitate subsequent classification and characterization analyses, including the identification of relationships with known archaeal viruses. The remaining potential proviruses will necessitate the development of more sophisticated analytical pipelines for their discovery. The identification of archaeal proviruses through MCP homology searches revealed a substantial number of previously undetected archaeal proviruses within archaeal genomes. This indicates a high diversity of archaeal viruses, underscoring the significance of exploring these hidden proviruses for uncovering the genetic material that has been designated "genetic dark matter" (64).

### Clustering classification of archaeal proviruses

The clustering approach of gene-sharing network was utilized to investigate the genetic relationships among the 38 proviruses, the archaeal (pro)viruses from IMG/VR4 (41), the ICTV 135 reference archaeal viruses, and 411 environmental archaeal (pro)virus genomic sequences (42–46).

**Fig 3:**
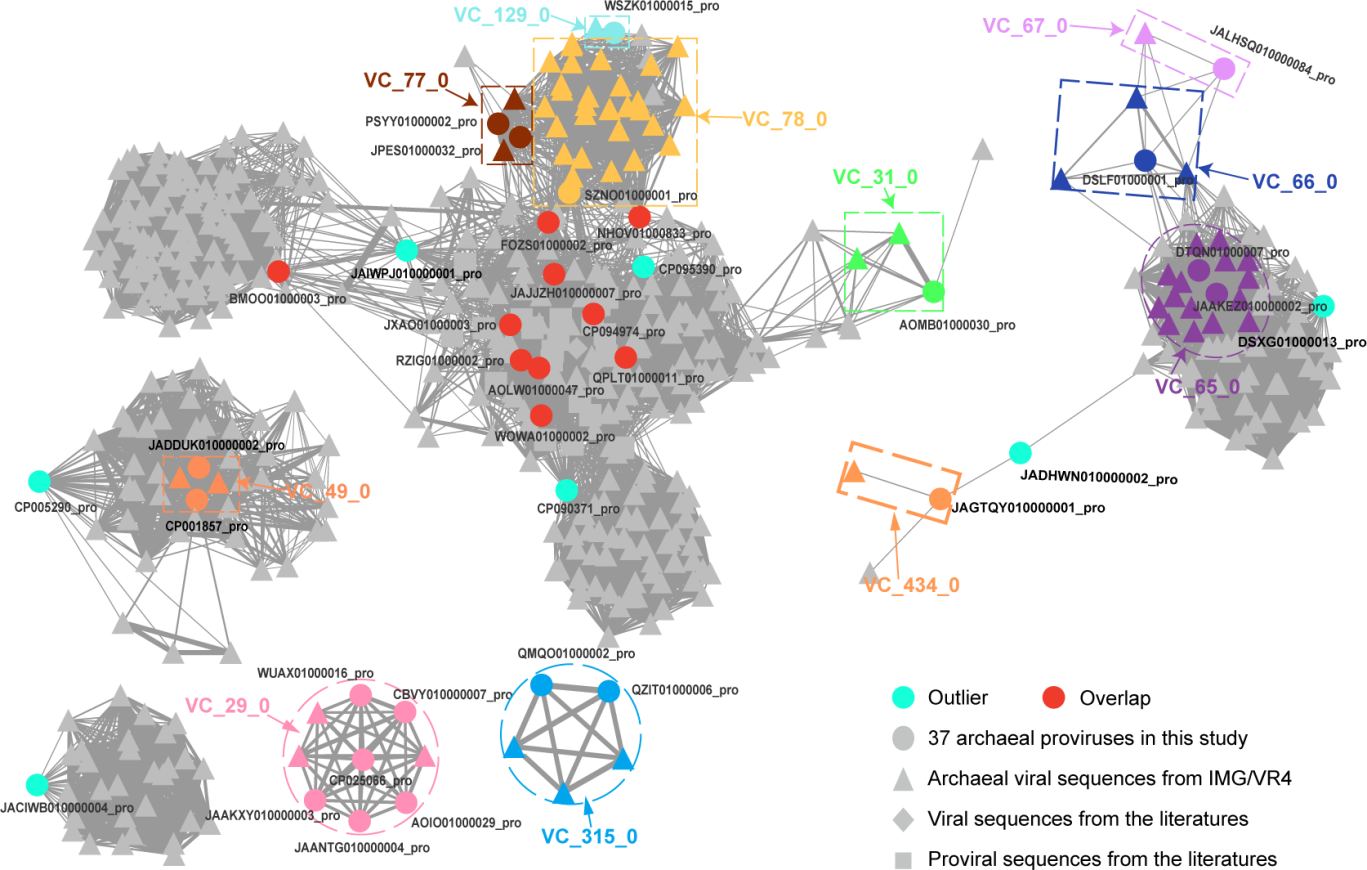
The proteome-based gene-sharing network of the 38 proviruses. Only the sequences that were connected to the proviruses are shown. Different shapes represent different viral sources: round for the 37 archaeal proviruses in this study, triangle for archaeal viral sequences from IMG/VR4, rhombus for viral sequences from the literatures, and square for archaeal proviral sequences from the literature. Different colors represent different viral clusters (VCs), overlaps, and outliers. VCs are individually marked with a circle or square.

**Fig 4:**
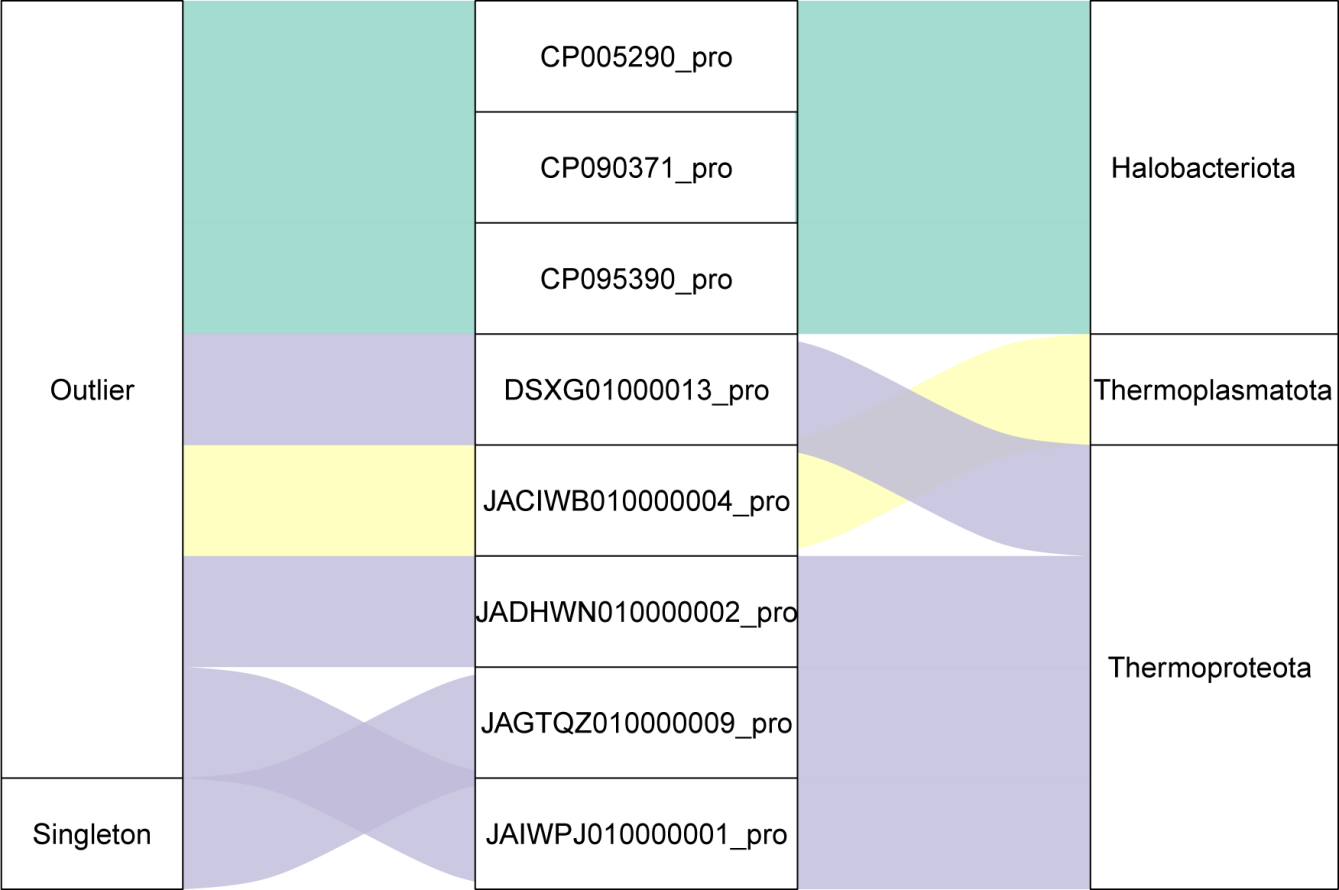
Correspondence between the proviruses clustered as outlier and singleton and their hosts. The first column lists the clustering levels of outlier and singleton in the vConTACT2. The second column shows the sequences’ name of the proviruses. The third column indicates the taxonomic ranks of the hosts of the proviruses at the phylum level.

Twenty proviruses (Fig. 3; Table S4) were grouped with archaeal viruses from the IMG/VR4 database (41), forming 11 viral clusters (VCs) that could be classified in the same genus or subfamily (40, 65). Ten proviruses exhibited shared core genes with multiple VCs and were clustered in an overlapping manner. Seven proviruses were identified as outliers of the VCs, and one provirus was classified as a singleton, indicating a markedly distant phylogenetic relationship to any known archaeal viruses. The hosts of these eight distantly related proviruses (seven outliers and one singleton) included three from halophilic archaea, one from the phylum *Thermoplasmatota*, and four from the class *Bathyarchaeia* of the phylum *Thermoproteota*, including the host of the singleton provirus (Fig. 4). The clustering results collectively emphasize the rich diversity of the 38 proviruses, including the discovery of unique viruses in both halophilic archaea and *Bathyarchaeia* archaea. Halophilic archaea exhibit a higher level of viral diversity (66), which aligns with the bias observed in archaeal databases due to the relative ease of cultivation of these organisms in laboratory settings. While some *Bathyarchaeia* archaeal (pro)viruses have been previously documented (67–70), a considerable number of novel proviruses remain to be discovered. This suggests that the viral diversity within the *Bathyarchaeia* archaea is exceptionally high and that there is significant potential for further investigation of the diversity, genomic features, and evolutionary impact of proviruses in *Bathyarchaeia*, a diverse and widespread group of archaea that play significant roles in various ecosystems, particularly in anoxic environments (71).

**Fig 5:**
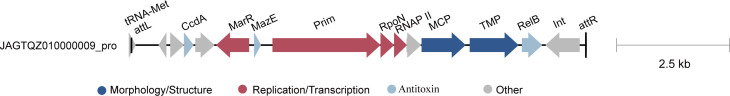
The genome map of the singleton provirus JAGTQZ01000009_pro. Arrows represent ORFs, and different colors indicate different functional categories of the annotated proteins.

The genome of the singleton provirus JAGTQZ010000009_pro contains three antitoxin genes: *CcdA* (ORF4), *MazE* (ORF7), and *RelB* (ORF14) (Fig. 5). In response to stress or adverse conditions, bacterial or archaeal cells may produce toxins with the objective of inhibiting growth or causing cellular damage (72). Antitoxins serve to neutralize the toxic activities produced by bacteria and archaea. In this manner, toxin–antitoxin (TA) systems provide assistance to the host in responding to stress. The presence of multiple antitoxin genes in a provirus may be indicative of a significant level of stress experienced by the archaeal cell, or alternatively, of an environment in which the cell is likely to encounter toxins (73). With regard to the structural genes, in addition to MCP, JAGTQZ010000009_pro also exhibited the presence of the tail length tape measure protein (TMP) (Fig. 5), which plays a role in chaperoning and directing the polymerization of tail components to form a tail of the appropriate length (74). The genes involved in replication and transcription, including DNA primase, sigma factor RpoN, RNA polymerase II (Pol II), and integrase, are present in JAGTQZ010000009_pro (Fig. 5), which is characteristic of temperate phages (75, 76). However, the provirus JAGTQZ010000009_pro has a restricted set of structural proteins and lacks the modules required for invasion and packaging, in contrast to other (pro)viruses. Furthermore, it contains a considerable number of antitoxin genes. It can be reasonably inferred that this provirus is defective and therefore unlikely to be capable of undergoing a lytic cycle. The concept of defective proviruses in archaea is less well documented in the scientific literature than that of viruses in bacteria and eukaryotes. Nevertheless, this is an area of growing interest within the field of virology. In extreme environments where archaea are frequently encountered, an understanding of the role of defective proviruses could provide insights into microbial community dynamics and resilience.

### Taxonomic classification of proviruses

**Fig 6:**
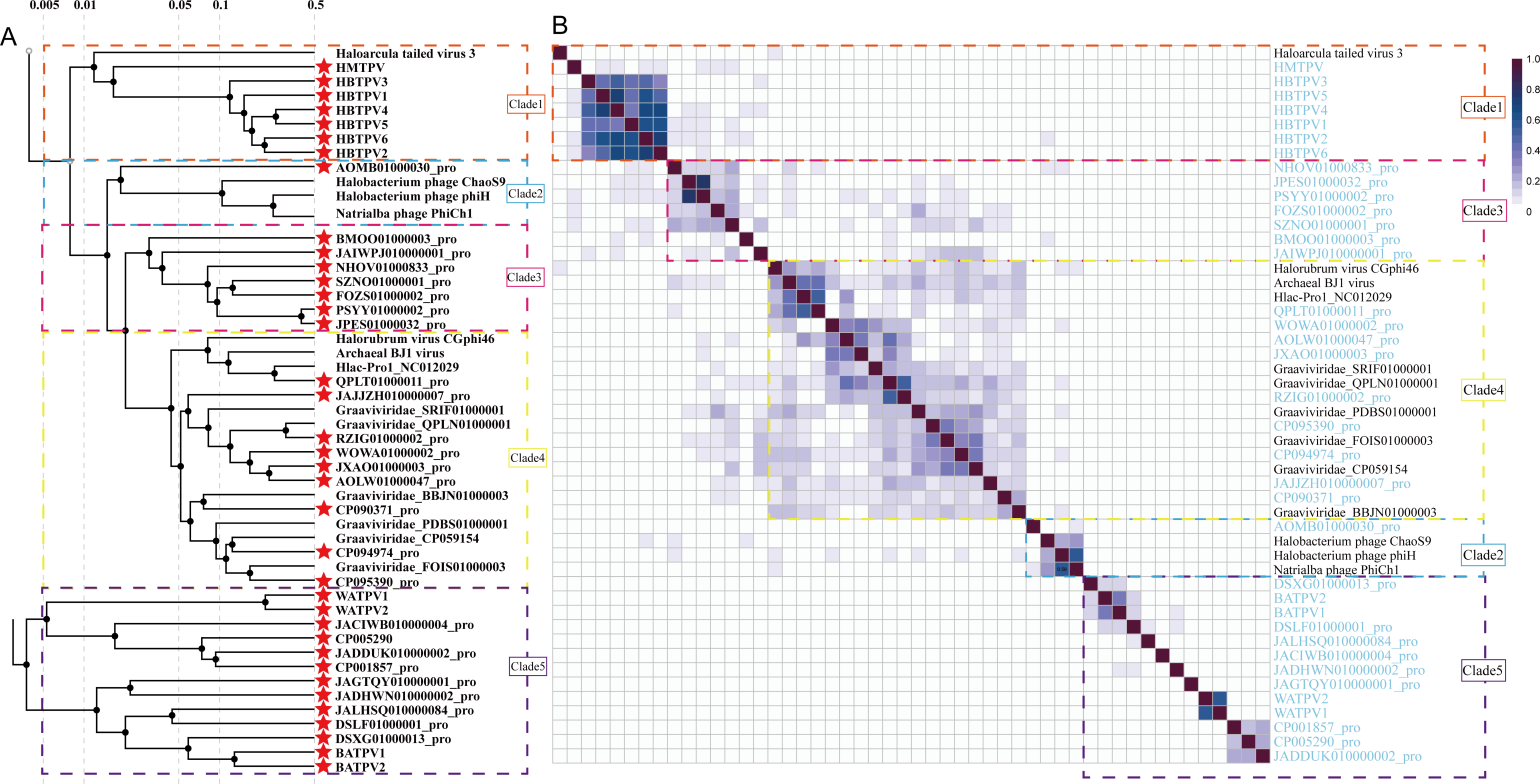
(**A**) The proteomic phylogenetic tree and (**B**) heatmap of orthologous fractions of the proviruses. The five clades are marked using the dashed lines. The proviruses in this study are indicated using the red stars ahead of their names (**A**) and in light blue (**B**).

To ascertain the taxonomic ranks of the 37 proviruses (with the exception of the potentially defective provirus JAGTQZ010000009_pro), a phylogenetic analysis of viral proteomes was conducted using ViPTree (58). In accordance with the prevailing standards for viral classification, members of the same genus are expected to exhibit a greater degree of protein homology, with at least 60% of their proteins sharing similarity. This proportion is reduced to 20% to 50% for members of the same family, while viruses from disparate families are anticipated to display a minimal level of protein conservation, with less than 10% of their proteins sharing homology (42). The results indicate that the proviruses were clustered into five distinct clades in proximity to related archaeal head-tailed viruses, and clades 1–4 exhibited a closer relationship with one another than with clade 5 (Fig. 6A).

**Fig 7:**
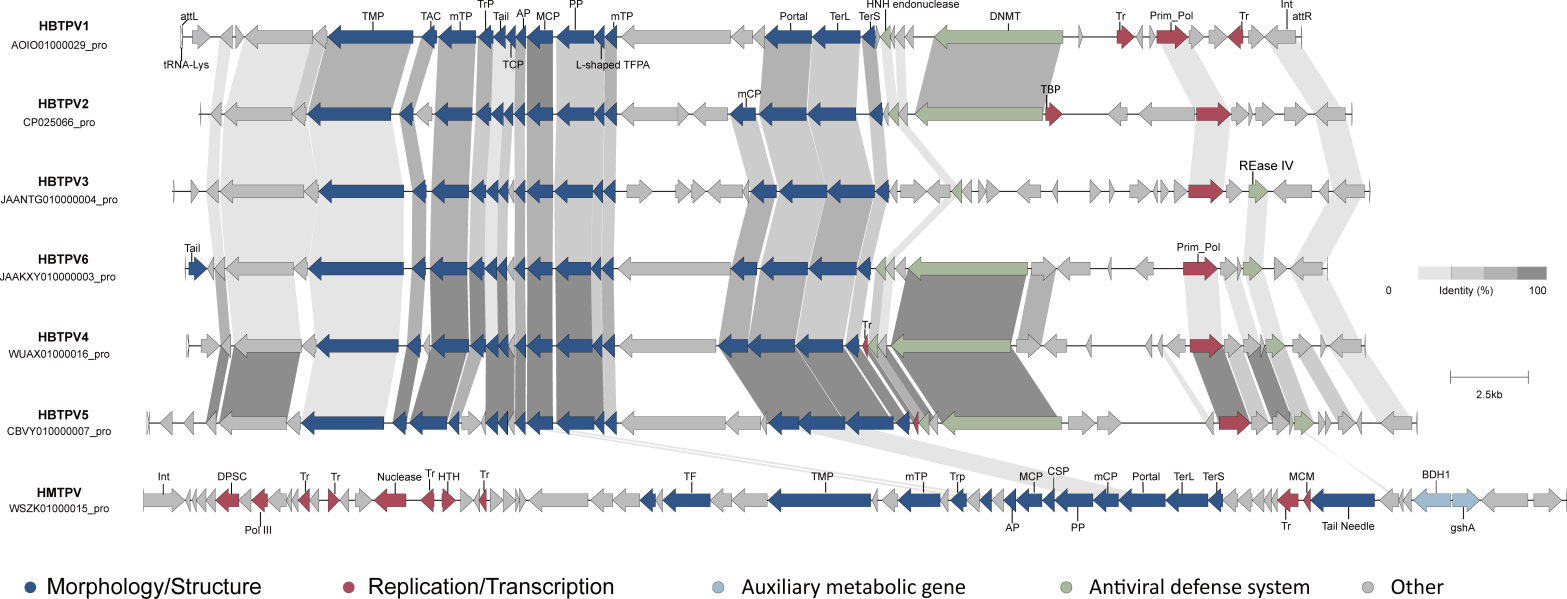
The genes shared in the clade 1 proviruses. Arrows represent ORFs, and different colors indicate different functional categories of the annotated proteins. All the shared proteins have >30% identity. The darker the linkage colors between the shared genes, the higher the shared similarity.

Clade 1 contained seven proviruses that were distantly related to the reference virus HTV3 (Fig. 6A). Six of these proviruses, named *Halobacteriales* tailed proviruses (HBTPVs), were found to be more closely related to each other (SG>0.05; OF >20%) (Fig. 6) and shared 18 genes, particularly those associated with morphology and structure (Fig. 7) (77). The only shared proteins between the HBTPVs and the seventh provirus (WSZK01000015_pro), designated the *Halomarina* tailed provirus (HMTPV), in clade 1, were the MCP, portal protein, adaptor protein, and one hypothetical protein (Fig. 7). It can be inferred that these six HBTPVs are members of a single family, whereas the HMTPV represents a distinct family.

In clade 2, provirus AOMB01000030_pro represents an early diverged lineage to three halophilic archaeal viruses (Fig. 6A). A limited number of homologous proteins are shared between the groups (SG <0.05; OF <10%) (Fig. 6), which indicates that this provirus may be classified as belonging to a novel family. Clade 3 comprises seven proviruses (Fig. 6). Five of the remaining proviruses (exclusive of BMOO01000003_pro and JAIWPJ010000001_pro) exhibit an OF between 20% and 80% in comparison to the viruses belonging to the *Graaviviridae* family (Fig. 6B), which indicates the potential affiliation of these proviruses with the aforementioned family. It is postulated that the BMOO01000003_pro and JAIWPJ010000001_pro are new viral families (SG <0.05; OF <20%) (Fig. 6). Nine proviruses were grouped with the viruses of the *Graaviviridae* family in clade 4 (SG >0.05; OF >20%) (Fig. 6), indicating a probable affiliation with the *Graaviviridae* family. It is important to note that the *Graaviviridae* family exhibits considerable evolutionary distance and diversity (42). Further investigation is required to elucidate the classification relationships within this family, particularly with the discovery of more closely related viruses. Clade 5 comprised 13 proviruses that exhibited a distant relationship to reference archaeal viruses (Fig. 6A). It is possible that they could be classified into nine viral families (SG <0.05; OF <20%) (Fig. 6). It is noteworthy that these viruses had a wide range of hosts, including *Bathyarchaeia*, *Woesearchaeales*, *Archaeoglobi*, and *Thermoplasmatota* (Table S3).

A proposal has recently been put forth that all archaeal tailed viruses (arTV) be classified into 14 families (42). Comparative genomics and host range analysis have revealed the significant diversity and evolution of arTV within the order *Caudovirales*. ArTV are evolutionarily related to tailed double-stranded DNA (dsDNA) bacteriophages in the class *Caudoviricetes*, with related proviruses found in *Nitrososphaeria* (formerly *Thaumarchaeota*) and *Aigarchaeota* (12, 19, 29, 78, 79). At the genomic level, arTV exhibit considerable diversity and minimal similarity to one another, with almost no resemblance to bacteriophages and eukaryotic tailed viruses. The considerable diversity and distinctness of arTV highlight the necessity of studying archaeal (pro)viruses as a discrete and distinctive group, which can facilitate a deeper comprehension of viral diversity and evolution.

### Genes involving the structure and replication of the proviruses

To investigate the potential biological traits of the 38 archaeal proviruses, we conducted gene functional annotation and classification. Of the 2,064 predicted ORFs, 23% (479) were grouped into COG functional categories (Fig. S1). The majority of the proteins were classified as mobilizing (20%), replicating (20%), and transcribing (14%), in keeping with the nature of viruses, which hijack the host replication and transcription machinery for growth. Genes associated with defense accounted for 6%, suggesting that viral and host interactions are a common occurrence.

The 38 proviruses were found to contain typical *Caudoviricetes* genes (Table S5) involved in the synthesis of capsid and tail structures, including HK97-like MCP, head maturation protease, portal protein, adaptor, minor tail protein, tail assembly chaperone, and tail completion protein. Additionally, genes associated with genome replication and packaging were identified, including DNA polymerase, minichromosome maintenance protein MCM, DNA primase, DEAD/DEAH box helicase-like protein, DNA polymerase sliding clamp, terminase large subunit, and terminase small subunit, as predicted by HHPred (36, 50). The provirus JAGTQY010000001_pro encodes a Holliday junction resolvase (HJR) (Table S5), which cleaves four-stranded DNA structures (holliday junctions) to produce two free double-stranded DNA molecules (80). The provirus JADHWN010000002 was found to contain both *HerA* and *TrmB* (Table S5). HerA is a DNA helicase that plays a pivotal role in the replication and packaging of viral genomes (81). TrmB has been demonstrated to regulate the expression of numerous genes in archaeal viruses, including those involved in processes such as replication, packaging, and host interactions (82). A primase–polymerase (Primpol) domain protein belonging to the PrimPol-PV1 supergroup was identified in 12 proviruses (Table S5). The primpol domain proteins have been identified in prokaryotic mobile genetic elements (MGEs), including (pro)viruses and plasmids, and are thought to play a significant role in the replication of archaeal viruses (83).

### Genes involving the defense of the proviruses

**Fig 8:**
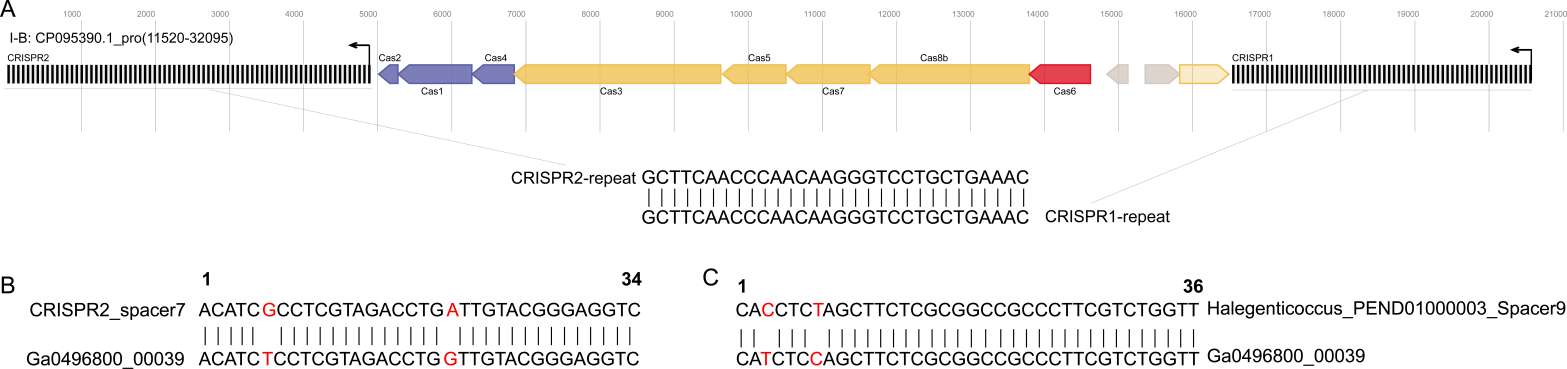
(**A**) Schematic presentation of the nested type I-B CRISPR–Cas system in the provirus CP095390.1_pro. The short black vertical line represents the CRISPR repeat, the sequence of which is shown below the schematic chart. The right-angle arrows indicate the transcription direction of the CRISPR array. The colored arrows indicate the Cas proteins. (**B**) Spacer–protospacer match between the provirus CP095390.1_pro (spacer seven in CRISPR 2) and the virus Ga0496800_00039 in IMG/VR4. (**C**) Spacer–protospacer match between the virus Ga0496800_00039 and its host of *Halegenticoccus*. The mismatched nucleotides are shown in red.

The proviruses possess various defense-related systems (Table S6). In particular, a nested type I-B CRISPR/Cas system was identified in CP095390_pro, with an evidence level of 4 (Fig. 8A). In contrast to the conventional configuration of CRISPR/Cas systems, this particular system is composed of two CRISPR arrays positioned between a Cas gene cluster, with both arrays and the Cas gene cluster exhibiting the same transcriptional orientation (Fig. 8A). The two arrays contain 74 and 61 spacers, respectively, and the repeat sequences are identical, with a length of 30 base pairs. Archaeal viruses have also been observed to contain CRISPR/Cas systems (84, 85). However, this nested structure has only been found in bacteria, archaea, and plasmids (86, 87). The nested CRISPR/Cas system is capable of discriminating against multiple PAM sequences, thereby enhancing its capacity to target a wider array of invaders (86). Based on spacer-protospacer matching (25, 53, 88, 89), the 135 spacers of CP095390_pro did not match its host. However, one spacer did match the virus (Ga0496800_00039) from the IMG/VR4 database (coverage = 100%; identity = 94.1%) (Fig. 8B) (41). Further examination of the data package containing Ga0496800_00039 revealed that this virus was targeted by a spacer from an archaeon of the *Halegenticoccus* genus in the *Halobacteriaceae* family (coverage = 100%; identity = 94.4%) (Fig. 8C). The host of CP095390_pro is also from *Halobacteriaceae*, which suggests that the nested type I-B CRISPR/Cas system in CP095390_pro likely functions as part of the host’s immune system, guarding against the invasion of foreign mobile genetic elements.

The provirus CP025066_pro contains the phage anti-restriction-induced system (PARIS) (Table S6), which represents a bacterial defense system against phages. This system triggers growth arrest when bacteria detect phage-encoded anti-restriction proteins (90). The provirus DSLF01000001_pro contains the helicase–methylase–ATPase (HMA) system, comprising three proteins with predicted domains of helicase (*HmaA*), m5c methyltransferase (*HmaB*), and ATPase (*HmaC*) (Table S6). This system has been identified in both archaea and bacteria, and its presence is consistent with that of other virus defense systems found in prokaryotes (52). The provirus JALHSQ010000084_pro possesses a type II R-M defense system (Table S6), which has been previously identified in bacteria and archaea as a means of combating foreign genetic materials (91, 92). This system employs two enzymes: a restriction endonuclease that cleaves DNA at specific sequences and a methyltransferase that modifies these sequences to prevent cleavage (93).

**Fig 9:**
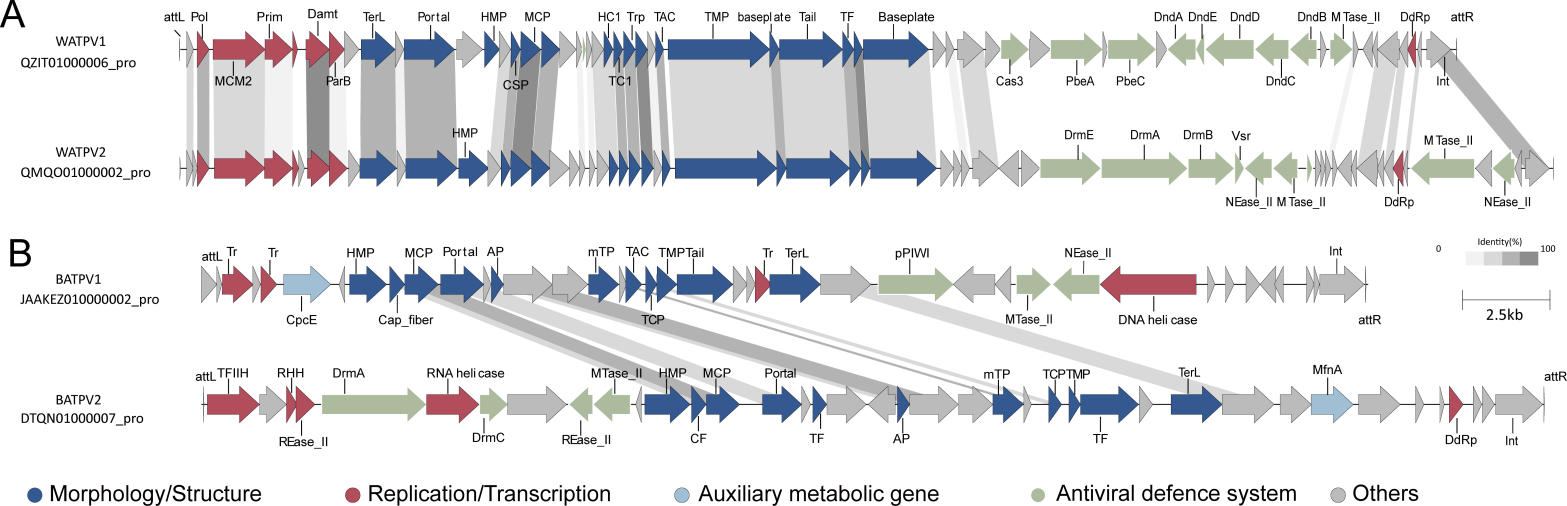
Colinear alignment maps of the genomes of WATPV and BATPV. Arrows represent ORFs, and different colors indicate different functional categories of the annotated proteins. All the shared proteins have >30% identity. The darker the linkage colors between the shared genes, the higher the shared similarity.

The proviruses QZIT01000006_pro and QMQO01000002_pro, named *Woesearchaeales* tailed proviruses (WATPVs), possess a rich antiviral defense system, including the class 2 Defense Island System Associated with Restriction–Modification (DISARM) (*DrmE*, *DrmA*, *DrmB*, and *MTase_II*), and DNA degradation (Dnd) (*PbeA*, *PbeC*, *DndA*, *DndE*, *DndD*, *DndC*, and *DndB*) (Fig. 9A). The DISARM system has been proposed as a novel R–M defense system and is prevalent in bacteria and archaea, exhibiting extensive antiviral capabilities (94).

The provirus DTQN01000007_pro, named *Bathyarchaeia* tailed provirus 2 (BATPV2), contains the genes of *DrmA* and *DrmC* (Fig. 9B), which are part of the DISARM system and likely play auxiliary roles in the DISARM system (94). The gene *pPIWI*, which is part of the Argonaute antiviral defense system (95), was discovered in the provirus JAAKEZ010000002_pro, named the *Bathyarchaeia* tailed provirus 1 (BATPV1) (Fig. 9B). To date, only two nucleic acid-guided defense systems have been identified: Ago and CRISPR–Cas systems. The *Sulfolobus islandicus* employs a three-protein system, comprising the short pAgo and its two genetically related proteins, Aga1 and Aga2, to mediate Abi responses and thereby achieve robust antiviral capabilities (96).

Collectively, these proviruses contain a substantial number of antiviral defense-related genes (Table S6) that impede the infection of other viruses, inhibit viral replication, and thereby safeguard the hosts from the intrusion of invading nucleic acids. This provides the foundation for the phenomenon of superinfection exclusion (Sie) (97), which elucidates the intricate interactions between (pro)viruses and their hosts.

### Auxiliary metabolic genes of the proviruses

Six auxiliary metabolic genes were detected across five proviruses (Table S7). Surprisingly, the phycocyanobilin lyase subunit alpha (CpcE) gene was found in the BATPV1 (JAAKEZ010000002_pro) of *Bathyarchaeia* archaea (Fig. 9B), a finding that has not yet been reported in any other viruses. The CpcE is an important enzyme involving the biosynthesis of phycobiliproteins, which are light-harvesting complexes primarily present in cyanobacteria and red algae (98). At present, the genus *Halobacterium* is the only known archaeon capable of photosynthesis (99). This is despite the presence of genes involved in photosynthetic processes, including those encoding bacteriochlorophyll A synthase, protochlorophyllide reductase, chlorophyll(ide) b reductases NOL/NYC1, NAD(P)H quinone oxidoreductase, photosystem I assembly protein Ycf3, and phycocyanobilin lyase, have been identified in *Crenarchaeota* (100) and *Heimdallarchaeota* (101).

**Fig 10:**
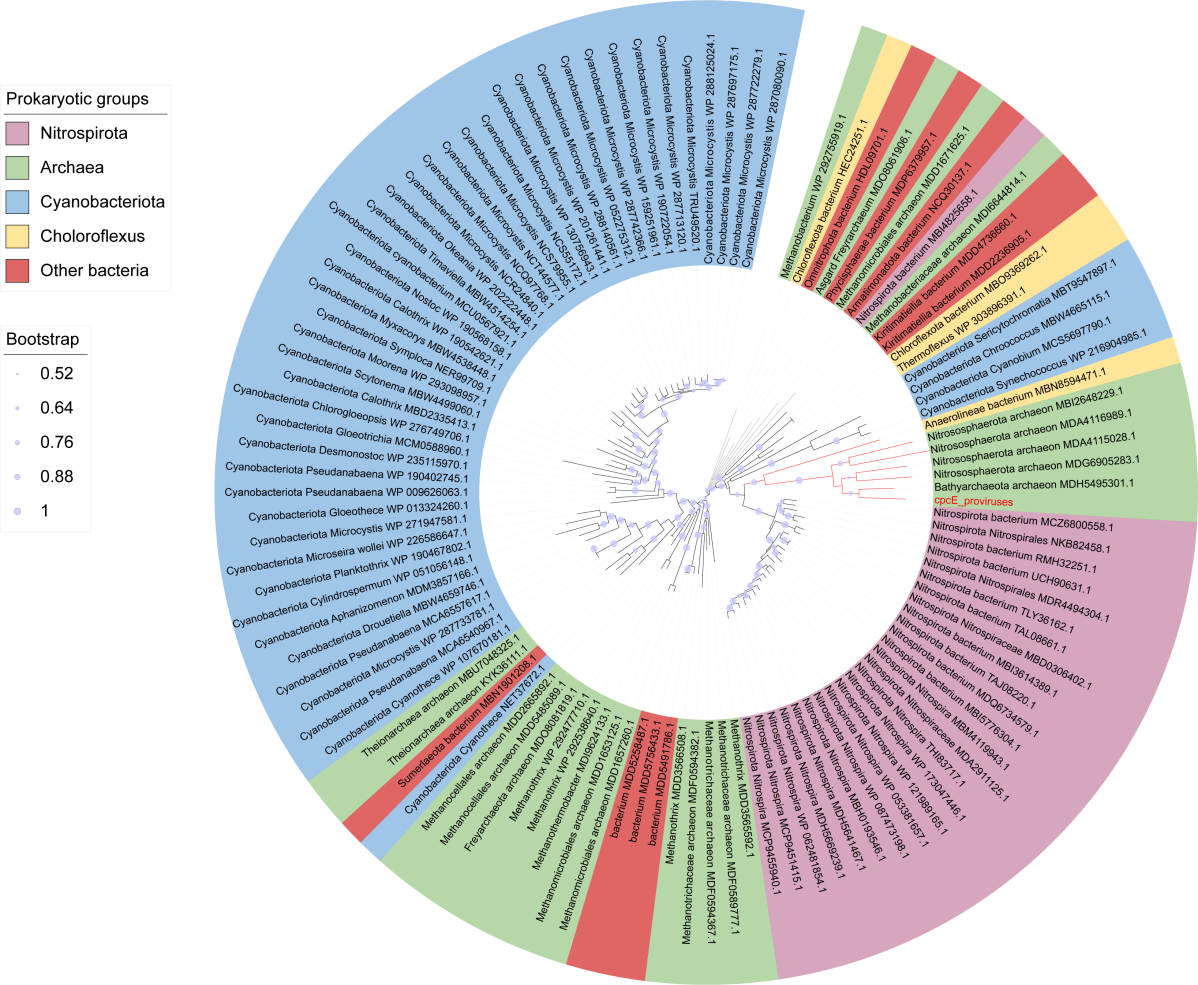
The maximum likelihood phylogenetic tree of the CpcE homolog gene. The red branches indicate that the CpcE homolog genes are closely related to those in the provirus. The different background colors indicate different prokaryotic groups.

The CpcE genes were found mainly in cyanobacteria, nitrospirotae, and some archaea (Fig. 10), which occupy analogous ecological niches. These genes exhibited a propensity for horizontal transfer between associated bacteria and archaea (Fig. 10). The provirus CpcE homolog gene was found to be phylogenetically grouped with that of the *Bathyarchaeota* archaeon (Fig. 10), to which the host of this provirus is affiliated. This indicates that the CpcE homolog gene was transferred horizontally between the *Bathyarchaeota* archaeon and archaeal proviruses. The CpcE homolog genes were derived from the *Nitrososphaeria* and could be traced back to the *Cyanobacteriota* phylogenetically (Fig. 10).

To ascertain whether the CpcE homolog gene in archaea has functions associated with photosynthesis, a KEGG annotation was conducted on the host of the provirus and another *Bathyarchaeota* archaeon that contains the CpcE gene (102, 103). The results indicated that neither of them contained the complete photosynthesis pathway, with the exception of a few related genes present in very small proportions (<0.1%) (Table S8). It is therefore unlikely that the CpcE homolog gene of the provirus and its host is involved in photosynthesis. However, it may be linked to other, as yet unknown, mechanisms (104, 105).

ORF34 in WOWA01000002_pro encodes sulfite oxidase, which catalyzes the oxidation of sulfite to sulfate, a less toxic compound (106). Notably, elemental sulfur serves as the electron donor in both aerobic and facultatively aerobic archaea (106, 107). It undergoes oxidation through sulfite and thiosulfate intermediates via a pathway involving sulfite:acceptor oxidases and has been demonstrated to integrate with the aerobic respiratory chain (108). The gene WSZK01000015_pro_64 is responsible for encoding glutamate–cysteine ligase EC 6.3.2.2, also known as GshA. GshA has been demonstrated to direct the synthesis and accumulation of γ-glutamylcysteine (γGC) in halophilic archaea (109), thereby contributing to the maintenance of an intracellular reducing environment. This γGC functions as a reducing agent for key reductases, thereby enabling the organism to resist oxidative stress, inhibit disulfide bond formation, and detoxify exogenous compounds (110). DTQN01000007_pro encodes the enzyme tyrosine decarboxylase EC:4.1.1.25, also known as mfnA. MfnA has been demonstrated to produce tyramine in *Methanocaldococcus jannaschii*, functioning as a coenzyme that reduces CO₂ into methane (111). The gene JAGTQY010000001_pro_10 encodes UDP-glucose 4-epimerase [EC:5.1.3.2], which catalyzes the reversible interconversion between UDP-glucose and UDP-galactose. Furthermore, it is an essential enzyme in the sugar nucleotide pathway (112).

The carriage of AMGs by these viruses has the potential to influence host physiology, survival, and adaptation to different environmental conditions. Further research is required to gain a comprehensive understanding of the intricate interactions between proviruses and their hosts, as well as the precise functions of these AMGs in the carbohydrate and amino acid metabolism of their hosts.

### Lytic potential of the proviruses

Twenty-six proviruses were predicted to be virulent using Phatyp (54), of which eight contained holin or endolysin genes (Table 1). Four proviruses were predicted to be temperate but had holin or endolysin genes (Table 1), indicating their capacity for replication and cell lysis (55, 56). As illustrated in Fig. 6, all of the proviruses identified in clade 4 were predicted to have lytic potential and classified within the family *Graaviviridae*. The *Graaviviridae* family encompasses viruses that infect archaea, particularly those that inhabit extreme environments. These viruses have the capacity to integrate into the host genome and remain dormant until triggered to enter a lytic cycle, wherein they replicate and cause cell lysis (44). For example, the Archaeal BJ1 virus (GenBank accession AM419438), a member species of the *Graaviviridae* family, was isolated from haloarchaeal strains and demonstrated to be a lytic virus. Moreover, evidence of integration has been observed in the case of the BJ1 virus (113). This adaptation confers a survival advantage in stable yet extreme environments. Taken together, 30 proviruses may lyse hosts and release viral particles under specific conditions, such as radiation or harsh environments, continuing to play a role in microbial communities. Meanwhile, there is a high likelihood that they will be induced and isolated in laboratory cultures.

**TABLE 1:** Predicting the lytic potential of proviruses

Accession no.	Length (bp)	Pred by Phatyp	Score
AOIO01000029_pro	36,084	Virulent	0.998265
AOLW01000047_pro	49,137	Virulent	0.999848
AOMB01000030_pro	41,349	Virulent	0.999867
BMOO01000003_pro	44,293	Virulent	0.753929
CBVY010000007_pro	40,868	Virulent	0.704037
CP001857_pro	33,648	Virulent	0.999589
CP005290_pro^*a*^	39,989	Temperate	0.999859
CP025066_pro	37,118	Virulent	0.999849
CP090371_pro	38,176	Virulent	0.999856
CP094974_pro^*a*^	59,467	Virulent	0.999866
CP095390_pro	62,752	Virulent	0.999865
DSLF01000001_pro^*a*^	38,584	Temperate	0.999857
DSXG01000013_pro	38,223	Temperate	0.978087
DTQN01000007_pro^*a*^	38,397	Virulent	0.999835
FOZS01000002_pro	40,261	Temperate	0.752539
JAAKEZ010000002_pro	33,358	Temperate	0.994198
JAAKXY010000003_pro	55,160	Virulent	0.999445
JAANTG010000004_pro	38,551	Temperate	0.99937
JACIWB010000004_pro^*a*^	22,121	Virulent	0.992033
JADDUK010000002_pro^*a*^	36,487	Virulent	0.995403
JADHWN010000002_pro	40,798	Temperate	0.999859
JAGTQY010000001_pro	55,009	Temperate	0.781648
JAIWPJ010000001_pro^*a*^	56,518	Virulent	0.999216
JAJJZH010000007_pro	41,968	Virulent	0.999865
JALHSQ010000084_pro	27,026	Virulent	0.999742
JPES01000032_pro^*a*^	40,384	Virulent	0.993022
JXAO01000003_pro	40,578	Virulent	0.999865
MTMI01000013_pro^*a*^	34,632	Virulent	0.99199
NHOV01000833_pro^*a*^	36,136	Virulent	0.923637
PSYY01000002_pro	40,766	Virulent	0.99982
QMQO01000002.1_pro^*a*^	55,455	Temperate	0.960029
QPLT01000011_pro	54,241	Virulent	0.99986
QZIT01000006	51,544	Virulent	0.999866
RZIG01000002_pro	65,939	Virulent	0.999867
SZNO01000001_pro	41,994	Temperate	0.964508
WOWA01000002_pro	35,464	Virulent	0.999858
WSZK01000015_pro^*a*^	45,795	Temperate	0.653216
WUAX01000016_pro	37,912	Temperate	0.999857
JAGTQZ010000009_pro	10,101	Temperate	0.99

^*a*^ Annotated with holin or endolysin genes.

### Conclusion

The study revealed that a multitude of archaea are harboring a vast array of proviruses. Specifically, 38 new provirus genomes were identified, originating from the following four main phyla: *Halobacteriota*, *Thermoplasmatota*, *Thermoproteota*, and *Nanoarchaeota*. All of the aforementioned proviruses were classified as belonging to the *Caudoviricetes* class and could potentially be grouped into 14 new families. The proviruses exhibited a variety of adaptation-related genes that counteract the immune systems of their hosts, suggesting a complex evolutionary history between viruses and archaeal hosts. The proviruses of *Bathyarchaeota* and *Halobacteriota* contain auxiliary metabolic genes, indicating that they employ distinct viral adaptation strategies in their hosts. These proviruses may serve as valuable reference viruses for further investigation into the enigmatic virosphere within archaea.

## Data Availability

The full genomes of the 38 proviruses are accessible in Table S3. The supplementary code for this article can be accessed online via the following link: https://github.com/Mr-Wornock/Archaeal-provirus.

## SUPPLEMENTAL MATERIAL

Figure S1 spectrum.00492-24-s0001.tif COG function category.

Supplemental tables spectrum.00492-24-s0002.xlsx Tables S1 to S8.

ASM does not own the copyrights to Supplemental Material that may be linked to, or accessed through, an article. The authors have granted ASM a non-exclusive, world-wide license to publish the Supplemental Material files. Please contact the corresponding author directly for reuse.
