# Supplementary figures and images for: Discovery and characterization of complete genomes of 38 head-tailed proviruses in four predominant phyla of archaea
Source: Microbiol Spectr. 2024 Nov 15;13(1):e00492-24. doi: 10.1128/spectrum.00492-24 (PMC11705971; doi:10.1128/spectrum.00492-24)

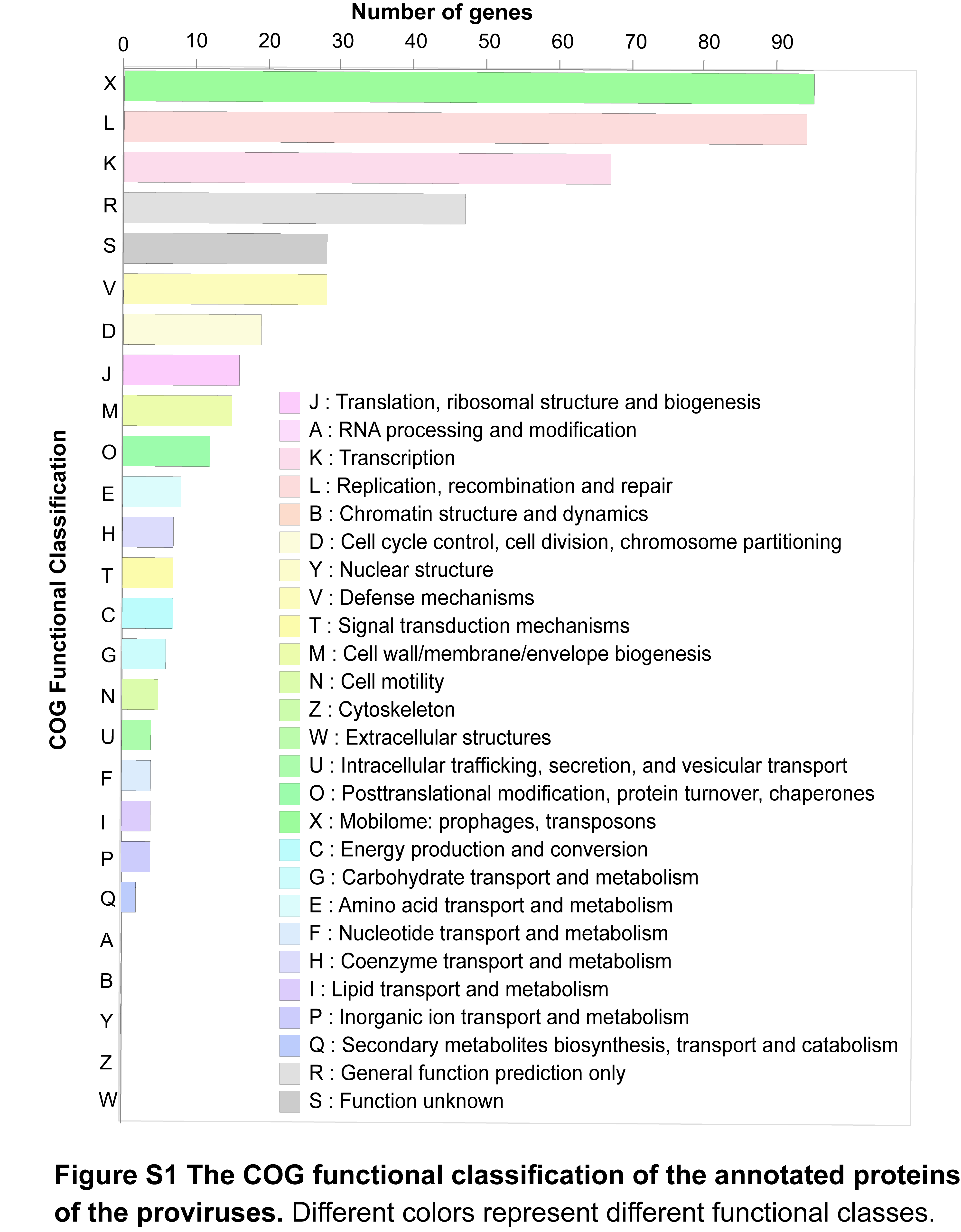

Supplement: Figure S1 — COG function category. [file spectrum.00492-24-s0001.tif]
